# A Clinical Lecture on Acute Pulmonary Phthisis

**Published:** 1852-04

**Authors:** David W. Yandell

**Affiliations:** One of the Consulting Physicians to the L. M. Hospital, of Louisville, Ky.


### WESTERN JOURNAL

OF

MEDICINE AND SURGERY.

APRIL, 1852.

### ART. I.—*A Clinical Lecture on Acute Pulmonary Phthisis.* By DAVID W. YANDELL, M. D., one of the Consulting Physicians to the L. M. Hospital, of Louisville, Ky.

*Gentlemen:* Acute pulmonary phthisis, or as it is more expressively called in popular language, "galloping consumption," is by no means a common form of the affection. Indeed, it is very rare. Tubercular consumption is essentially a chronic disease. The mean term of its duration is stated by Sir James Clarke to be from nine to eighteen months, while Andral fixes it at two years; and cases are recorded which have lingered forty and even fifty years.

Of the many individuals suffering from phthisis received into the Louisville Marine Hospital since my connection with that institution in 1848, all have had the disease in its chronic form. In three hundred and fourteen cases reported in the works of Louis and Bayle, only twenty-four, or about eight per cent., were subjects of acute phthisis—two died within one month, ten within two months, and twelve within three months. The tables of both Louis and Bayle, however, are calculated on cases which occurred in hospital practice, where, I am satisfied, the disease is more frequently than elsewhere seen in the acute form. You will find before you have been many years in the practice, that pulmonary phthisis affecting the poorer classes who are necessarily deprived of the many comforts and conveniences that so much tend to retard the progress and mitigate the intensity of disease, is one thing; and pulmonary phthisis, when it attacks the affluent, is another and quite a different thing.

Andral and Dr. Walshe recognise three varieties of acute phthisis, while Sir James Clarke admits only two. Andral bases his divisions upon local symptoms,—as being like or unlike those of chronic phthisis. Dr. Walshe founds his on the diffeıent anatomical states of the disease; and Sir James Clarke on the character of the morbid process and the constitution of the patient. Each of these several varieties is not without its practical utility as I think you will presently see.

The first divisions of Andral and Walshe are alike, in both being characterized by local symptoms and pathological lesions identical with those observed in chronic phthisis. Here, however, the analogy ceases.

In Andral's second variety the symptoms are not only remarkable for the rapidity with which they succeed one another, but also, and in a still greater degree, as not being those which usually denote the existence of tubercles in the lungs; and in his third variety there is an entire absence of all local symptoms whatsoever. Yes; singular as it may appear, particularly to those of you who have been taught to lay great stress upon physical diagnosis, there have been patients in whom the rapid development of pulmonary tubercle was announced by no local symptom—in whom auscultation could detect nothing but the healthiest respiratory murmur, and percussion elicited no abnormal sounds—in whom the breathing was scarcely interfered with, and who had but slight cough and no expectoration.

I have remarked that in the first variety, as described by Dr. Walshe, there was nothing peculiar save the rapidity of its course—that the symptoms, signs, and anatomical lesions are the exact counterpart of those observed in chronic phthisis. In the second and third varieties of the affection, however, both symptoms and physical signs are more or less peculiar, and are far less characteristic than you might at first suppose, or than is desirable. What these are will presently be pointed out.

We come now to the two varieties of acute phthisis as delineated by Sir James Clarke. This gentleman, as I have told you, thinks that in one form "the short duration of the disease appears to depend chiefly on its violence, or the activity of the morbid process; and in the other, on the feeble powers of the constitution, which sink under the pulmonary disease long before it has reached the degree in which it usually proves fatal." The symptoms of either variety are often obscure and the diagnosis difficult; both are observed chiefly in young persons—in three instances out of four according to Andral, and the last more frequently in females than in males according to Sir James Clarke. The first, which is much the most striking variety, frequently follows in the wake of some one of the acute exanthemata, and is said occasionally to select its victims from among individuals in whom there had never been suspicion of the existence of tubercles—individuals without hereditary taint or vice of constitution, of previously robust and even vigorous health. In this form of the affection the symptoms succeed each other with extreme rapidity; the fever runs high; the perspiration is profuse, the cough violent, the expectoration and diarrhea copious, the exhaustion great. As far as my own observation goes, however I am not inclined to believe that acute phthisis ever occurs in individuals who are entirely free from the scrofulous diathesis. Dr. Graves refers every form of consumption to one common origin, and that the strumous habit. The patient Ben, a free colored man, whom many of you had an opportunity of examining, died within five weeks of the time when he first presented either rational or physical signs of phthisis. He had always been regarded by those who knew him as a perfectly healthy man, and yet he had sprung from a stock deeply tainted with tubercles; has lost brothers with consumption, and had in all probability been himself long the subject of tubercular deposit at the summit of his left lung. Those of you who live in the South, and have seen much of its slave population have perhaps seen similar cases—cases in which tubercles were deposited in the lungs to an extraordinary extent within a few weeks without there having been hemoptysis, cough, emaciation, or any of the symptoms that are usually considered the constant forerunners and inseparable attendants of this disease. But I venture to say that none of you have ever seen cases of this description except in persons of that debilitated state of the constitution termed the scrofulous habit; or in those who have a hereditary predisposition to phthisis which proves too often an inalienable birth right.

Ben was thirty-six years old, married, temperate, active, industrious; of fine size, and athletic form, and had he not been descended of scrofulous parents, and lost brothers of phthisis between the ages of eighteen and thirty-five years—the period stated by Hippocrates to be that at which this disease is most common, we should never have thought that he was likely to perish in this way. Furthermore, until within a few weeks of his last attack, when he had purchased his freedom, Ben was the property of a humane and considerate master, who had always provided him with comfortable quarters, good food, and ample clothing; and knowing his hereditary tendencies had carefully protected him from undue exposure and too much work. He had been healthy, and presented every mark of a sound man until one month before his last sickness. About this time, meeting him on the street, I was struck with the change in his gait from its usual quick, and active step to the slow and languid locomotion of an invalid. And here, gentlemen, let me remark that you may often gather from the voice, gait, and aspect of an individual while still going about and attending to his ordinary affairs, that he is the subject of disease. As has been remarked by Dr. Latham, "there are certain popular tokens of disease which all the world is acquainted with," and those that I have mentioned as being present in our patient are of them. To return—Ben told me that he had been "under the weather" for a few days—had been troubled with constipation and severe pain in his back, two very common things with negroes, particularly the latter—that he had wandering pains about the chest, cephalalgia, and no appetite; was languid, his breath short, and his sleep unrefreshing. A severe epidemic influenza prevailing at the time, which was attended by a train of symptoms not unlike those just mentioned, I directed him to take what had proved very beneficial under the same circumstances in other persons, to wit: A Dover's powder and two blue pills on going to bed, and a dose of castor oil the following morning. He did so, and was benefited. Two weeks after this interview I was sent for to see him. He had been confined to his room four days with what some of you will remember he called "a heavy cold," and which proved to the poor fellow a very fatal one. Although we found him sitting up dressed, his appearance was that of a sick man. His skin was hot and dry, pulse 100, quick; respiration 30, attended with some pain at the summit of the left lung where the motion of expansion was deficient; cough frequent, expectoration copious, frothy, composed of serum and mucus; tongue coated with a white fur; bowels obstinately constipated, urine high colored, scanty; thirst, anorexia. His sleep was interrupted so much by coughing that he could not be said to rest at all. He was very weak; complained of severe pain in the back and head, and a sense of extreme oppression about the chest —a feeling, which had increased greatly within the preceding twenty-four hours. This was on the 26th day of December. I remember that one of you who was with me at the time, thought before we examined our patient's chest, that his disease was inflammation of the parenchyma of the lungs. Careful auscultation and percussion, however, told, though obscurely, another story; and thereby added another to the countless instances where physical signs have come in to modify and correct the impression made by the rational symptoms. Here, now, is what physical exploration revealed, and though its revelations were by no means as satisfactory as we might have wished, they are still sufficiently so, I think, to enable us by exclusion, that most beautiful process of reasoning as applied to the science of diagnosis—to approximate if not altogether arrive at the truth: Inspection and mensuration disclosed nothing special; the hand when applied to the chest detected a very slight increase in the vocal vibration of the left side, and a diminution of chest motion in the left clavicular and infra-clavicular regions. The resonance under percussion was perfect, save over the superior third of the left lung, and there it was but slightly impaired. The respiration was weak over the same regions and exaggerated over corresponding points of the opposite side. Between the third and fifth ribs of the left side, the expiratory sound was perceptibly prolonged. Such were the lights afforded us by a carefully conducted physical examination. They are meagre, perhaps, as far as positive evidence goes, and yet when seen in a negative point of view they become of very great significance. While they cannot be said to have established the existence of tubercles, they have clearly shown that there was not inflammation either of the covering, lining membrane, or parenchyma of the lungs —that there was neither pleuritis, bronchitis, or pneumonitis. Being unable to ascertain the existence of either of these diseases, and satisfied from an examination of the other organs of the body that they were not the seat of the lesion, I concluded that the train of phenomena detailed must depend upon the irritation produced by the development of tubercles.

Notwithstanding this opinion, I was at a loss to account for certain of the symptoms, or at least, for the want of correspondence between those that were rational and those that were physical. Why, in this case, was dyspnea one of the prominent symptoms when in other cases, absolutely similar in respect to the nature of the alteration and the suddenness of its development, the breathing remains entirely free? Why was there such an almost entire absence of positive physical signs?

Gentlemen, there are few things more difficult to explain than this wide difference of phenomena produced by one and the same cause. It is however, what Andral calls "a primary fact," one with which we constantly meet in the study of disease, and from which we deduce one of the most valuable principles of pathology—one upon which the whole art of diagnosis rests, to wit: "that from one and the same lesion, all things else being equal, the most different symptoms may result, according to the individual." Individual predisposition or idiosyncrasy alone can explain this inconstancy in the connexion between cause and effect.

I have so often seen in children, suffering from acute pulmonary disease, difficulty of breathing and even a sense of suffocation, the result of a loaded and distended state of the bowels, that I believed much was due to the same cause in Ben; and in view of this directed him to take castor oil and turpentine, and to use a purgative injection. But here, gentlemen, let me caution you against the use of purgatives, as a class, in the treatment of acute phthisis. Purgation is peculiarly obnoxious because of the danger of exciting the tuberculizing process in the intestines, and should never, I think, be resorted to except in a few rare cases.

In addition to the indication intended to be fulfilled by the oil, etc., I deemed it necessary to attempt the relief of the local congestion of the lung by the application of cups over the seat of the disease, and to allay constitutional and pulmonary irritation by diaphoretics and sedatives. Ben, however, would not hear to either cups or leeches and contented himself with taking an occasional pill of Dover's powder and tartar emetic.

I ordered a full mercurial and opiate at bed time, and being myself taken sick the next morning, did not see him again for five days. His wife reported on the following afternoon that his breathing had been much improved after his bowels were acted upon, and that he had passed a comfortable night. The changes that had been effected within the five days were most striking and dispiriting. I report them as the result of a protracted exploration of the patient's chest by Dr. Breckinridge and myself. There was perceptible depression both above and below the left clavicle; chest motion was very manifestly exaggerated save in the region already mentioned, where it was almost null. Dullness on percussion distinct and even tubular. An occasional humid crackling and thin metallic mucous ronchus at the extreme summit of the lung, and blowing respiration with the *cogged wheel* rhythm of the respiration, which latter condition Dr. Walshe states he has *never* met with except in tuberculous patients. The voice and cough sounds were exaggerated.

I neglected to say that the conditions just mentioned were present with almost equal distinctness over corresponding regions of both the anterior and posterior parts of the thorax, and that the respiration was sharply puerile over the superior third of the right lung.

The rational symptoms were not less noticeable or significant. The expectoration had changed in quantity and quality; was larger in amount and muco-purulent in character. The cough was less paroxysmal, more constant and hacking. The pulse was 115, quick, and jerking—the result of excessive irritation. For two days there had been chills and sweating—the former slight, the latter copious. This peculiar condition of the pulse and sharp febrile excitement, are regarded by Dr. Gerhard as of great importance in the diagnosis of tuberculous disease of an acute kind. He indeed designates them as peculiar to tubercles. There was constipation, and I hardly need add anorexia and thirst. Emaciation and loss of strength kept pace with the other changes.

Dr. B. agreed with me that the case was one of acute phthisis. What then was to be done? Such, ordinarily, is the difficulty of diagnosticating acute consumption, that there are few diseases about the treatment of which more has not been written. The lancet, used with the utmost circumspection—local abstractions of blood in doubtful cases; counter irritation, at a certain distance from the chest; diaphoretics and sedatives, and abstinence from purgation; antimony exhibited only in cases where the arterial action is excessive, and even then with extreme caution, because of the tendency to failure of strength, and but little more that is hopeful or encouraging in the use of mercury, are almost the sum total of the therapeutic means recommended by the authors who have written most extensively on this subject.

Meagre indeed are they, and all that one can hope to accomplish by them is, in some degree, to hasten the transformation of acute into chronic phthisis.

Such had been the rapidity of the emaciation and general failure of the vital functions, that although I believed there was considerable tendency to pneumonia, yet I, did not regard the almost certain danger of prostration, likely to follow venesection as outweighed by the very questionable advantage to be derived from the use of the lancet. The perverseness of the patient obliged us to relinquish all thought of local bleeding. Nor did I feel myself warranted in the exhibition of tartar emetic in contra-stimulant doses, for the same reason that decided me against general blood-letting. According to my own experience, tartarized antimony is far too powerful a medicine, however guardedly given and closely watched, to be administered in cases where the depression is as decided and the tendency to great and even disastrous prostration so manifest as in the present instance.

Rejecting the lancet and tartar emetic, and compelled to forego both cups and leeches, I attempted the immediate relief of the dyspnea, which had again become urgent, by the application to the chest of a large mustard sinapism, and directed tincture of opium and ether. I was desirous of making the smallest quantity of opium answer for the relief of both the dyspnea and cough; for as the disease progresses it is generally necessary to increase the quantity, and vary the preparation, "as it often becomes in the last stages the chief solace of the patient amidst his multiplied sufferings." A few leeches to the sternal notch would have done more, perhaps, to allay the cough than opium or any other narcotic whatever, but these, for the reason that I have already stated, were refused.

A well regulated diet being considered the best means of controlling the hectic fever of phthisis, I counseled the patient in this particular; and, in addition, told him to sponge during the hot stage his hands and feet in tepid vinegar and water, and prescribed bark for the relief of the cold fit, which, you know is often the most distressing part of the febrile paroxysm. I gave the bark in combination with sulphuric acid, and, in addition, cautioned the patient against the use of much warm fluid, particularly towards night, hoping by these means to diminish the copious perspirations from which he had suffered. For the abdominal congestion, which was evidently considerable, I prescribed a mercurial alterative.

At the end of four days, not only had this plan of treatment produced no sensible amelioration whatever, but in the meanwhile a frequent and exhausting diarrhea had supervened, which threatened to prove more rapidly destructive than any previous symptom. I resolved upon a change of measures, and instead of the opium, bark and acid, directed the patient to use that most invaluable agent in the chronic form of the disease, the cod-liver oil. This substance, you are aware, is stated by Dr. Walshe more rapidly and effectually to induce improvement in the general and local symptoms of phthisis than any other known agent, and many of you have been witnesses both in my hospital and private practice of its, I had almost said marvelous effects in this affection. It is true that I never administered it in such cases as were attended with decided intra-thoracic inflammation, and for that reason hesitated to prescribe it for Ben. But all other agents of established efficacy had been tried without any mitigation whatever of suffering—our patient was fast sinking into the grave, and while there were perhaps, contra-indications to the use of the oil, under the circumstances, yet I deemed it greatly preferable to any other remedy. I ordered it to be taken in doses of a drachm twice daily, a quantity that I have found quite sufficient in the beginning. I know physicians who give it by the wine-glassful several times a day, but for my own part I have never seen any good, and often observed ill effects follow this practice. One ounce and a half in the twenty-four hours, I have found to accomplish the full amount of good that it appears is effected by the oil. The results of its administration to Ben were manifest within thirty-six hours, and altogether peculiar. It relieved the cough without diminishing the expectoration, abbreviated the duration and lessened the copiousness of the sweats without allaying the fever—checked the diarrhea without arresting the emaciation, not disagreeing with the stomach while it did not improve the appetite: In a word, alleviating to a truly remarkable extent the intensity of most of the prominent symptoms without appearing, even in the slightest degree, to stay the progress of the disease.

Those of you who saw the case will remember, that I called your attention to the quantity of the expectoration which was truly enormous. Now, expectoration when excessive, often constitutes almost as serious a drain upon the system as a diarrhea, and its diminution forms one of the indications of cure in the advanced stages of many cases of both acute and chronic chest disease. I have seen the best results follow the use of chalybeates, especially sulphate of iron, and Griffith's steel and myrrh mixture, in persons in the last stages of phthisis, in whom there was little gastric irritation or fever, and although this patient suffered from both the last mentioned symptoms, yet I thought it proper to make trial of some preparation of this nature, and gave Griffith's mixture. I do not think that its administration produced any sensible diminution in the expectoration, nor do I, on the other hand, believe that it increased the fever.

Counter irritants, you are aware, have long occupied a chief place among the various remedies which have been employed in phthisis. They should however be employed only after the inflammatory action has been subdued—

used before this time, they frequently increase the evil by exciting irritation in the system. Sir James Clarke thinks that they are especially liable to do so in consumptive patients, and declares that the prevailing error in the use of blisters is their too early application. Do not lose sight, gentlemen, of one very important clinical fact, to wit: that blisters are never beneficial when they excite much pain and irritation; and that the less uneasiness they give, the less distress they occasion, and the greater discharge they produce, the more is the benefit derived from them. Such was the heat of the surface and general irritability of the system of our patient, that I ventured upon the use of but a single blister, which was attended by an amount of suffering that prevented my repeating the experiment.

Louisville, Ky., March, 1852.

